# Viral hepatitis in female sex workers using the Respondent-Driven Sampling

**DOI:** 10.1590/S1518-8787.2017051006540

**Published:** 2017-06-20

**Authors:** Marcos André de Matos, Divânia Dias da Silva França, Megmar Aparecida dos Santos Carneiro, Regina Maria Bringel Martins, Lígia Regina Franco Sansigolo Kerr, Karlla Antonieta Amorim Caetano, Raquel Silva Pinheiro, Lyriane Apolinário de Araújo, Rosa Maria Salani Mota, Marcia Alves Dias de Matos, Ana Rita Coimbra Motta-Castro, Sheila Araújo Teles

**Affiliations:** I Faculdade de Enfermagem. Universidade Federal de Goiás. Goiânia, GO, Brasil; II Secretaria Municipal de Saúde. Goiânia, GO, Brasil; III Instituto de Patologia Tropical e Saúde Pública. Universidade Federal de Goiás. Goiânia, GO, Brasil; IV Departamento de Saúde Comunitária. Universidade Federal do Ceará. Fortaleza, CE, Brasil; V Instituto Federal de Educação, Ciência e Tecnologia. Campus Goiânia Oeste. Goiânia, GO, Brasil; VI Departamento de Estatística e Matemática Aplicada. Universidade Federal do Ceará, Fortaleza, CE, Brasil; VII Centro de Ciências Biológicas e da Saúde. Universidade Federal de Mato Grosso do Sul. Campo Grande, MS, Brasil

**Keywords:** Sex Workers, Hepatitis B, epidemiology, Hepatitis C, epidemiology, Seroepidemiologic Studies

## Abstract

**OBJECTIVE:**

To estimate the prevalence of hepatitis B virus and C virus infections and their genotypes and analyze the risk factors for the markers of exposure to hepatitis B virus in female sex workers in a region of intense sex trade.

**METHODS:**

This is a cross-sectional study performed with four hundred and two female sex workers in Goiânia, Brazil. Data have been collected using the Respondent-Driven Sampling. The women have been interviewed and tested for markers of hepatitis B and C viruses. Positive samples have been genotyped. The data have been analyzed using the Respondent-Driven Sampling Analysis Tool, version 5.3, and Stata 11.0.

**RESULTS:**

The adjusted prevalence for hepatitis B virus and C virus were 17.1% (95%CI 11.6–23.4) and 0.7% (95%CI 0.1–1.5), respectively. Only 28% (95%CI 21.1–36.4) of the participants had serological evidence of vaccination against hepatitis B virus. Being older (> 40 years), being single, having a history of blood transfusion and use of cocaine, and ignoring the symptoms of sexually transmitted infections were associated with positivity for hepatitis B virus (p < 0.05). We have detected the subgenotype A1 of hepatitis B virus (n = 3) and the subtypes of hepatitis C virus 1a (n = 3) and 1b (n = 1).

**CONCLUSIONS:**

We can observe a low prevalence of infection of hepatitis B and C viruses in the studied population. However, the findings of the analysis of the risk factors show the need for more investment in prevention programs for sexual and drug-related behavior, as well as more efforts to vaccinate this population against hepatitis B. The genotypes of the hepatitis B virus and C virus identified are consistent with those circulating in Brazil.

## INTRODUCTION

Approximately 400 million persons are infected with hepatitis B virus (HBV) or hepatitis C virus (HCV) worldwide, which cause acute and chronic hepatitis, cirrhosis, and liver cancer. These infections result in one million deaths annually^a^.

These viruses are transmitted through parenteral and sexual contact^a^, though the role of sexual transmission in HCV infection remains unclear^43^. In addition to having multiple partners, many female sex workers (FSW) are also illicit drug users. The propensity of these women to transmit HBV and HCV to their partners and vice versa cannot be ignored^1,26,38^.

Hepatitis B may be prevented by vaccination, but not hepatitis C^a^. In Brazil, hepatitis B vaccination has been offered for free for children and adolescents, as well as risk populations, for over 15 years^32^.

In the country, the practice of prostitution in itself is not illegal, but the activities that sustain this practice are considered as criminal acts^6^. It is estimated that 1% of the Brazilian female population aged 15–49 years are sex workers^31^. This means approximately half a million of FSW at high risk to transmit and acquire sexually transmitted infections^b^. However, most investigations involving FSW have been limited to HIV^4,8,25^. Data on the extent of hepatitis B and hepatitis C infections are still scarce and those that exist use convenience sampling that do not represent the population as a whole^23,30,39,41^.

Respondent-driven sampling (RDS) is a network-based method for sampling hard-to-reach populations (such as drug users) that produces asymptotically unbiased population estimates^14^. This method has been used mainly in HIV biological and behavioral surveys among illicit drug users, men who have sex with men, and sex workers^33^. Using this methodology, we estimated the prevalence of HBV and HCV and their genotypes, and analyzed the risk factors for markers of HBV infection among FSW in a region of intense sex trade in Brazil.

## METHODS

### Study Population and Sampling Method

This cross-sectional study was carried out among FSW in Goiânia, a medium-sized city with approximately 1.2 million inhabitants in the Midwestern region of Brazil, from May 2009 to June 2010. Goiânia is a focal point for the sex trade in Brazil^c^. The FSW were defined as women who exchange sexual services for money. The eligibility criteria were women who exchanged sex for money within the last 30 days. Exclusion criteria were being younger than 18 years of age or self-reporting as transgendered.

In this paper, we analyzed data from participants included in a previous study about HIV epidemiology among FSW in Goiânia^2^. Data were collected using RDS. This methodology has been widely used in investigations among men who have sex with men, illicit drug users, and sex workers because of difficulties to reach these populations using population-based sampling techniques. The RDS is a form of referral sampling, and it uses the social networks of the target populations by peer referral methods for recruitment. The recruitment process starts with a non-random selection of key members of the target population denominated as “seeds” that receive a pre-established number of recruitment coupons for distribution to members of their social network. If the persons recruited by the “seeds” are eligible, they are included in the study, and they receive coupons for subsequent peer referral. Thus, based on the Markov chain model, if the peer recruitment occurs from a sufficiently large number of recruitment waves, the dependence of the final sample on the initial convenience sample (“seeds”) is reduced^10,31^.

In this study, seven “seeds” were selected during May and June 2009. Of them, four meet their clients on the streets in different regions of the city, and the other three in nightclubs, brothels, and erotic movies. Their age ranged from 28 to 53 years old and their education level ranged from none to secondary level. Two were white, four brown and one black. After obtaining the informed consent and completing the interview, the seeds received three coupons to recruit other FSW who they knew by name, were aged 18 years or more, who they had encountered in the last 30 days, and who lived in Goiânia. The recruits also received the same number of coupons and invited another three FSW under the same conditions as before. This procedure was repeated until the desired sample size was achieved. As an incentive, each participant received six public transportation tickets for participating and two more transportation tickets for each successfully recruited FSW.

A sample size of approximately 380 FSW was calculated considering a 95% confidence interval, 80% power, effect design of 2.0^38^, and anti-HIV of 5.6%^29^.

### Data Collection and Laboratory Tests

Data were collected using a face-to-face structured questionnaire addressing sociodemographic data and risk factors for hepatitis B and C infections. Knowledge about the symptoms of sexually transmitted infections (STI) was evaluated by answering the following questions correctly: “Is burning pain during urination a symptom of STI? Are genital ulcers/sores symptoms of STI? Is itchy skin a symptom of STI?”.

After the interview, 10 ml of peripheral blood were collected from each FSW to test for the presence of markers of HBV (HBsAg, anti-HBc total, and anti-HBs), and anti-HCV by enzyme-linked immunosorbent assay (ELISA). Anti-HCV positive samples were re-tested for confirmation using a line immunoassay (INNO-LIA HCV Ab III; Innogenetics, Ghent, Belgium).

The HBV DNA was detected in HBsAg positive samples and the Pre-S/S region of the HBV genome was amplified using a semi-nested PCR assay^32,33^. Nucleotide sequences of the amplified region (S region) were determined by direct sequencing using a BigDye Terminator 3.1 cycle sequencing kit (applied Biosystems, Foster City, CA, USA). Sequences were aligned and edited using SeqMan II v.5.01 (DNASTAR), Clustal W, and BioEdit. To identify mutations in the S region, the deduction of amino acids was performed from the nucleotide sequencing using the MEGA 4 software. Anti-HCV positive samples were submitted to HCV RNA detection by nested PCR using primers that target the 5’-UTR region^11^. A line probe assay was used to determine the genotype of the isolates according to the procedure described by the manufacturer (Inno-LiPA HCV II, Innogenetics, Ghent, Belgium).

### Data Analysis

Data were analyzed using the Respondent-Driven Sampling Analysis Tool version 5.3 (RDSAT v.5.3) and Stata 11.0. The “seeds” were not included in analysis. We used three questions to measure the network size of each participant and the resulting weighting: ‘‘How many female sex workers do you know?”, “How many female sex workers do you know personally and who you have met in the last 30 days in Goiânia?”, “Considering all female sex workers that you know, how many of them are younger than 18 years?”.

Descriptive analyses and calculated population estimates with their confidence intervals were used for all the variables studied. The FSW who were only anti-HBs positive were excluded from the association analyses. Univariate and multivariate analyses using unconditional logistic regression were used to estimate predictors of HBV exposure. Variables with p-value < 0.20 were included in the model. As there is no consensus on the best statistical method to use in more complex analyses such as logistic regression using RDS sampling, we used the individual weights calculated by RDSAT and exported them to modeling data using Stata^15^. The significance level used in the tests was 5%. In a multivariate analysis, variables used to measure knowledge on STI were recoded, and “no” and “do not know” were interpreted to mean risk.

### Ethics Statement

The study has been approved by the Research Ethics Committee of the Universidade Federal de Goiás (Protocol 001/09). All women received pre and post-test counseling. The results of the serological tests were delivered personally in private places with presentation of the identity card or other official document. Participants who were positive for HCV and HBV were forwarded to the Reference Center for Diagnosis and Therapy in HIV/AIDS in the city of Goiânia.

## RESULTS

A total of 1,206 coupons were issued and 410 returned. Of those, 402 FSW were eligible and accepted to participate in the study. The median number of waves by seed was nine (range: 4–14) and the median of recruits was 63 (range: 13–92). The key variables age and education reached equilibrium at wave seven and two, respectively.

The characteristics of the participants are shown in Table 1. Most women were 30 years or younger and single. Only one fourth were white. Half of the women had less than 10 years of education, and approximately one third had already worked as sex workers in other cities in Brazil and abroad. Streets, night clubs, and bars were the favorite places to meet clients.

**Table 1:** Characteristics of female sex workers in Goiânia, Midwestern Brazil.

Characteristic	%^a^	%^b^	IC95%^b,c^
Age (years)			
< 21	14.4	13.8	9.4–19.8
21–25	26.4	31.7	24.5–40.3
26–30	23.9	25,2	18.3–32.6
31–35	14.2	12.2	7.1–16.3
36–40	8.5	5.8	3.2–8.8
> 40	12.7	11.3	5.4–18.3
Education level (years)			
≤ 4	10.4	9.7	6.3–15.1
5–9	41.0	40.6	32.2–47.3
10–12	45.5	47.4	40.4–56.9
> 12	3.0	2.3	0.4–2.4
White	24.6	27.3	20.5–35.4
Single	68.2	67.1	60.2–75.2
Works as female sex worker in another city	46.8	37.6	30.8–45.5
Place where she meets clients			
Street	32.3	25.0	10.1–43.2
Nightclub	30.6	41.0	28.4–53.7
Bar	24.9	27.7	15.4–41.8
Brothel	6.0	5.5	0.2–17.1
Erotic movies	10.0	3.8	0.4–11.6
Other	10.2	5.3	0.2–17.1
Average amount earned in the last day of work (US$)			
75.01–350.00	23.9	22.4	13.3–32.5
25.01–75.00	21.9	25.0	17.2–32.2
15.01–25.00	29.2	24.8	17.7–32.7
3.50–15.00	24.9	27.7	17.3–39.9

^a^ Unadjusted.

^b^ Adjusted by RDSAT.

^c^ 95% confidence interval.

The overall prevalence of HBV adjusted by RDSAT was 17.1% (95%CI 11.6–23.4), and 1.6% (95%CI 0.1–4.7) were infected because they were HBsAg positive. Only 28% (95%CI 21.1–36.4) of the women were positive for anti-HBs alone, suggesting previous vaccination against hepatitis B. Most of them (85.3%) were aged 30 years or less.

Four women were HCV positive. The adjusted prevalence of HCV was 0.7% (95%CI 0.1–1.5). All of these four women were older than 30 years. Three worked on the streets and were cocaine users. One reported previous blood transfusion.

Table 2 presents the univariate analysis of risk factors for HBV infection, in which age > 40 years, low education level, being single, meeting clients on the streets, no suggestion of condom use by both persons in the last sexual encounter, obtaining male condom exclusively in the workplace, cocaine use, previous blood transfusion, and ignoring STI symptoms (burning pain, genital ulcers/sore, itching) were associated with HBV exposure (p < 0.05). These variables besides age of first sexual intercourse and number of partners who did not pay for sex were included in the multivariate analysis, and the following variables remained independently associated with HBV positivity (Table 3): age [adjusted odds ratio (adjOR): 1.13], previous blood transfusion (adjOR: 3.16), cocaine use (adjOR: 4.54), being single (adjOR: 3.80), ignoring burning pain during urination as symptom of STI (adjOR: 2.57), and ignoring genital ulcers/sores as symptom of STI (adjOR: 3.47).

**Table 2:** Univariate analysis showing variables associated with markers of exposure to hepatitis B virus in female sex workers. Goiânia, Midwestern Brazil.

Variable	Total^a^	HBV positive^b^		Odds ratio	95%CI^c^	p

		%	95%CI^c^			
Age						
≤ 25	91	17.0	5.6–41.4	1.00	-	< 0.001
26–30	76	11.9	4.2–29.2	0.66	0.28–1.58	
31–40	82	27.1	15.1–43.7	1.82	0.88–3.75	
> 40	44	41.5	22.9–62.9	3.47	1.53–7.87	
Education level (years)						
≤ 4	39	37.3	20.8–57.3.	3.16	1.35–7.41	0.009
5–9	119	25.1	15.4–38.0	1.78	0.97–3.28	
≥ 10	135	15.8	6.8–32.7	1.00	-	
Single						
No	97	14.6	7.7–26.0	1.00	-	0.028
Yes	196	25.9	16.4–38.3	2.05	1.10–3.83	
Age of first sexual intercourse						
7–14	88	27.4	15.0–44.5	1.00	-	0.051
15–17	154	16.6	8.4–30.2	0.53	0.27–1.02	
18–30	50	29.2	13.5–52.2	1.09	0.50–2.39	
Meets clients on the street						
No	192	16.8	8.9–29.3	1.00	-	0.003
Yes	101	33.1	21.6–47.0	2.47	1.39–4.402	
Average amount earned last day of work (US$)						
75.01–350.00	58	23.6	7.4–54.5	1.00	-	0.283
25.01–75.00	62	21.5	9.7–41.2	0.47	0.22–1.02	
15.01–25.00	96	15.5	8.1–27.5	0.70	0.32–1.54	
3.50–15.00	76	28.1	16.2–44.1	0.79	0.37–1.71	
Partners who did not pay for sex (last week)						
≥ 1	133	16.9	7.5–33.9	0.61	0.34–1.09	0.114
0	159	25.2	16.3–36.9	1.00	-	
Always uses condom with clients						
Yes	267	20.9	13.7–30.5	1.00	-	0.325
No	23	30.5	10.4–62.3	1.61	0.67–3.87	
Use of condom with last client						
Yes	287	21.7	14.7–31.0	1.00	-	1.000
No	4	22.9	2.5–77.5	1.20	0.12–11.70	
In the last sexual intercourse the condom use was suggested by both						
Yes	81	13.6	6.6–26.0	1.00	-	0.039
No	206	24.9	15.9–36.8	2.12	1.04–4.31	
Use of condom with steady partner in the last sexual intercourse						
Yes	89	16.6	8.4–30.1	1.00	-	0.227
No	201	22.5	13.8–34.6	1.486	0.80–2.77	
Obtains male condom exclusively in the workplace						
No	225	25.4	16.9–36.4	3.77	1.44–9.88	0.003
Yes	64	8.0	3.0–19.9	1.00	-	
Alcohol use in the last month						
No/Only once a month	95	21.5	12.2–35.0.	1.00	-	0.983
At least three times a week	103	21.0	10.1–38.7	0.97	0.49–1.95	
Everyday	95	22.8	12.0–38.9	1.08	0.52–2.26	
Cocaine use^d^						
No	222	18.7	12.6–26.9	1.00	-	0.042
Yes	71	31.6	13.4–57.8	1.95	1.05–3.60	
Tattooing/Piercing						
No	127	22.5	14.4–33.2	1.00	-	0.886
Yes	166	21.1	11.4–35.8	0.93	0.53–1.63	
Previous blood transfusion						
No	249	19.1	12.0–29.1	1.00	-	0.004
Yes	44	47.3	29.8–65.5	3.63	1.58–8.32	
Is burning pain during urination a symptom of STI?						
Yes	195	16.2	9.9–25.3	1.00	-	0.003
No	29	28.1	10.9–55.5	2.02	0.83–4.90	
Do not know	69	35.9	18.0–58.8	2.90	1.54–5.48	
Are genital ulcers/sore symptoms of STI?						
Yes	210	15.3	9.4–24.0	1.00	-	< 0.001
No	27	23.2	8.5–49.4	1.67	0.73–3.81	
Do not know	56	46.1	24.6–69.2	4.73	2.41–9.28	
Is itchy skin a symptom of STI?						
Yes	206	20.1	12.9–29.9	3.25	0.95–11.10	0.001
No	24	7.2	1.5–28.0	1.00		
Do not know	63	37.0	18.3–60.7	7.60	2.09–27.58	

STI: sexually transmitted infection

^a^ Unadjusted.

^b^ Adjusted by Respondent-Driven Sampling Analysis.

^c^ 95% confidence interval.

^d^ Only one injection cocaine user; different denominators in the results represent missing data, which may have occurred from participants not answering certain questions.

**Table 3:** Multivariate analysis of the association between the variables studied and prevalence of hepatitis B virus. Goiânia, Midwestern Brazil.

Variable	Adjusted odds ratio*	95%CI
Age (years)	1.13	1.08–1.19
Previous blood transfusion	3.16	1.15–8.69
Cocaine use	4.54	2.01–10.28
Single	3.80	1.69–8.54
Ignoring burning pain during urination as a symptom of STI	2.57	1.14–5.78
Ignoring genital ulcers/sores as a symptom of STI	3.47	1.58–7.58

STI: sexually transmitted infection

* Adjusted by age, education, being single, age of first sexual intercourse, meeting clients on the streets, no suggestion of condom use by both persons, obtaining male condom exclusively in the workplace, cocaine use, previous blood transfusion, ignoring symptoms of sexually transmitted infections.

The HBV DNA was detected in all HBsAg samples in which the HBV genotype A (subgenotype A1) was identified. All had the mutation T131N in the S region. Of the anti-HCV positive samples, HCV RNA was genotyped as genotype 1, subtypes 1a (n = 3) and 1b (n = 1).

## DISCUSSION

To our knowledge, only Kassak et al.^16^ have used the RDS methodology to investigate viral hepatitis among Lebanese FSW. Therefore, this study represents the first epidemiological report on viral hepatitis among FSW in South America using the RDS methodology. This investigation provides information concerning HBV and HCV infections among FSW in Goiânia, a region of intense sex trade in Central Brazil. Using the RDS methodology, we reached FSW in multiple locations and urban neighborhoods.

Most FSW were young, single, nonwhite, and had low education. These characteristics were similar to those found in FSW recruited in ten cities in Brazil^6^, and in another capital of the Midwest region^8^.

The overall prevalence of HBV of 17.1% (95%CI 11.6–23.4) found among FSW was slightly higher than that estimated in the general Brazilian population aged 20–69 years (11.6%; 95%CI 10.7–12.4)^d^. However, it was lower than that observed by Lurie et al.^21^ among FSW prior to the availability of the hepatitis B vaccine in the country (39%; 95%CI 35.2–43).

Curiously, the prevalence of HBV found among FSW in Goiânia was similar to those observed by Pando et al.^36^ in Argentina (14.4%; 95%CI 11.8–17.6), Leon et al.^19^ in Bolivia (13.1%; 95%CI 7.8–21.2), and Camejo et al.^3^ in Venezuela (13.8%; 95%CI 9.7–18.9), suggesting a common HBV distribution among FSW in these countries.

Concerning the data of Kassak et al.^16^, these authors have not found any case of HBV exposure among FSW. However, it is noteworthy that they have recruited only 103 women and most of them were from other countries. Therefore, these women might not be representative of the Lebanese FSW as a population.

Almost all women vaccinated against HBV were aged 30 years or less, and they very likely had benefited more from the Brazilian policy of HBV vaccination among adolescents than from the policy of vaccination of high risk populations, suggesting gaps in the Brazilian strategies for the prevention of hepatitis B in target populations.

Three women were HBsAg positive and they were infected with HBV subgenotype A1, which circulates in Brazil^26^. Moreover, all of them had T131N mutations in the S region. Interestingly, this mutation has been found in patients infected with genotype A or G, and has been involved in immune escape^40^. In this study, one HBsAg positive woman reported previous hepatitis B vaccination. However, given the small number of cases, further investigations are desirable to evaluate the real role of this mutation in these women.

Four FSW were HCV positive, and they were infected with HCV subtypes circulating in Brazil (1a and 1b). Furthermore, the prevalence of HCV among FSW that we have estimated was close to the values reported in other FSW populations, in which the use of illicit injection drugs was infrequent or null^1,18^, indicating that this virus is not efficiently transmitted by heterosexual intercourse.

In this investigation, blood transfusion was found as a predictor of HBV, though screening for both HBsAg and anti-HBc markers in Brazilian blood banks has been in place for more than 20 years^e,f^, and most FSW reported blood transfusion after 1993 (26/44). However, the residual risk (RR) of hepatitis B in Brazil is still high compared to developed countries^17^, and nucleic acid testing (NAT) for HBV is optional in blood banks.

A quarter of the FSW investigated reported cocaine use, and we found this behavior to be a predictor of exposure to HBV. In addition, all but one of the FSW infected with HCV reported cocaine use. The overlap between illicit drug use and prostitution has been recognized as a public health problem, increasing the prevalence of HIV and other STI among FSW and their partners. Many FSW enter the sex work to support their drug addiction, or they become drug addicts to bear the sexual work^5,13,22^. In general, they have more clients than those who do not use illicit drugs and they practice more unsafe sex^7,25^. Furthermore, the sharing of items such as straws and pipes for sniffing or smoking (crack cocaine) may favor viral transmission in this population^9,33,35^.

Interestingly, being single was a predictor of exposure to HBV. These women reported increased consumption of alcohol and illicit drugs compared to those who were not single (data not shown). In fact, these behaviors have been related to unsafe sex^34,42^.

Genital ulcers/sores, itching, and burning pain during urination are classical symptoms of sexually transmitted infections. We used these symptoms to evaluate the knowledge on STI among the FSW. We have found that ignoring genital ulcers/sore and itching were predictors of exposure to HBV. These findings reinforce the assumption that less knowledge on STI leads to risk behaviors^43^ and, consequently, more opportunities to acquire these infections*.* In fact, a multi-level intervention combining the social-structural and clinical prevention of STI/HIV carried out among sex workers improved the consistent condom use with regular clients^20^.

Although the indirect variables “obtaining male condom exclusively in the workplace” and “suggestion of condom use by both” did not reach statistical significance following the multivariate analysis, these findings suggest the FSW studied have social vulnerabilities for STI. Condom use is the cornerstone of HIV prevention and condoms are distributed for free to FSW in the Brazilian public healthcare services. However, social stigma has been a barrier for this population to access health services^14^ and our findings suggest the need to make them available in their workplace to promote safe sex.

The disagreement in the negotiation of condom use may be a risk factor for STI. Clients who refuse to use condoms during sexual intercourse are more susceptible to the risks of unsafe sex, which in turn increases the chances of both parties acquiring sexually transmitted infections, including hepatitis B.

Limitations of the study include self-reports of sexual behaviors that can over or underestimate the chances of HBV positivity. The probability-based population survey is the gold standard for obtaining epidemiologic data; however, for hard-to-reach populations, such as the FSW, this represents a hard task. Using the RDS method, we have recruited FSW from a range of settings including streets, nightclubs, bars, brothels, erotic movie theaters, and elsewhere. In addition, we have attempted to select “seeds” with different characteristics to produce a well-networked and diverse sample.

In conclusion, the prevalence of HBV and HCV infection is low among FSW in Goiânia, Central Brazil. However, we have found a high frequency of FSW susceptible to HBV older than 30 years, in addition to the identified risk factors observed, which indicate that preventive interventions are needed for both sexual and drug-related behaviors, and more efforts should be made to vaccinate FSW against hepatitis B, mainly those older than 30 years. Finally, this study also demonstrates social vulnerabilities to sexually transmitted infections among these women, and potential for increased transmission of STI. These findings provide a starting point to address target interventions to prevent STI, including viral hepatitis B, in this high-risk population.

Soon after the end of the study, our research group performed several education actions in partnership with non-governmental organizations and health managers with the female sex workers, including visits with distribution of educative folders and condoms and the offering of hepatitis B vaccine in their workplace^27^.

## Footnotes

a World Health Organization. Prevention & control of viral hepatitis infection: framework for global action. Geneva; 2013 [cited 2015 Jun 1]. Available from: http://www.who.int/csr/disease/hepatitis/GHP_framework.pdf

b UNAIDS. The Gap Report: beginning of the end of the AIDS epidemic. Geneva; 2014 [cited 2016 mar 20]. Available from:http://www.unaids.org/en/media/unaids/contentassets/documents/unaidspublication/2014/UNAIDS_Gap_report_en.pdf

c Ministério Público do Estado de Goiás. Relatório do Centro de Apoio Operacional da Infância, Juventude e Educação. Goiânia; 2009 [cited 2016 Dec 6]. Available from: http://www.mp.go.gov.br/portalweb/hp/8/docs/relatorio_da_investigacao_casa_de_shows.pdf

d Pereira LMMB, Ximenes RAA, Moreira RC, coordenadores. Estudo de prevalência de base populacional das infecções pelos vírus das hepatites A, B e C nas capitais do Brasil. Recife: Universidade Federal de Pernambuco; 2010 [cited 2016 Dec 6]. Available from: http://www.aids.gov.br/sites/default/files/anexos/publicacao/2010/50071/estudo_prevalencia_hepatites_pdf_26830.pdf

e Ministério da Saúde (BR). Portaria nº 721, de 9 de agosto de 1989. Aprova normas técnicas para coleta, o processamento e a transfusão de sangue, componentes e derivados, e dá outras providências. *Diário Oficial União*. 11 ago 1989 [cited 2015 Jun 1]; Seção 1:13643-50. Available from: http://www.anvisa.gov.br/legis/portarias/721_89.pdf

f Ministério da Saúde (BR). Portaria 1376, de 19 de novembro de 1993. Normas técnicas para coleta, processamento e transfusão de sangue, componentes e hemoderivados. *Diário Oficial União*. 2 dez. 1993 [cited 2015 Jun 1]; Seção 1:18405-415. Available from: http://www.anvisa.gov.br/legis/portarias/1376-93.pdf
